# Carbon Sequestration, Plant Cover, and Soil Health: Strategies to Mitigate Climate Change

**DOI:** 10.3390/plants14233610

**Published:** 2025-11-26

**Authors:** Leonor Deis, Francesca Fort, Qiying Lin-Yang, Pedro Balda, Alicia Pou

**Affiliations:** 1 Facultad de Ciencias Agrarias, Fisiología Vegetal, Universidad Nacional de Cuyo, Mendoza M5500, Argentina; 2 Oenological Technology Group (TECNENOL), Department of Biochemistry and Biotechnology, Facultad de Enología, Universidad Rovira i Virgili, Sescelades Campus, C/Marcel·lí Domingo, 1, 43007 Tarragona, Spain; mariafrancesca.fort@urv.cat (F.F.); qiying.lin@urv.cat (Q.L.-Y.); 3 Department of Food and Agriculture, Scientific and Technology Complex, Universidad de La Rioja, c/Madre de Dios, 51, 26006 Logroño, Spain; pedro-jose.balda@unirioja.es; 4 Instituto de Ciencias de la Vid y del Vino—ICVV (CSIC, UR, GR) Finca La Grajera, 26007 Logroño, Spain; alicia.pou@icvv.es

**Keywords:** soil organic carbon, sustainable viticulture, carbon balance, *Vitis vinifera*

## Abstract

Climate change, driven largely by increasing atmospheric CO_2_ from fossil fuel combustion and soil carbon losses caused by unsustainable land use, threatens agricultural productivity and ecosystem services. Viticulture, developed mostly in Mediterranean and temperate regions, is particularly vulnerable by rising temperatures, decreasing precipitation, and soil degradation. Nevertheless, vineyards also offer opportunities to mitigate climate change by increasing soil organic carbon (SOC) and plant biomass. This review analyzes current scientific evidence on the impact of soil management practices in vineyards. Implementing strategies such as the use of cover crops, organic amendments, the incorporation of pruning residues, and reduced tillage can significantly contribute to carbon sequestration and soil health. Sequestration rates vary widely, from 2.8 to more than 11 Mg CO_2_ ha^−1^ year^−1^, depending on climate, soil type, and practices used. Average carbon sequestration rates for crops with minimum or reduced tillage range between 2.5 and 5 Mg CO_2_ ha^−1^ year^−1^, increasing to 7 and 7.5 Mg CO_2_ ha^−1^ year^−1^ when organic fertilizers are used. Uncertainties in the economic balance, initial costs, and weak political incentives hinder the adoption of sustainable agricultural strategies, highlighting the need for further research into expanding their application. These strategies also improve microbial activity, nutrient cycling, and resilience to abiotic stresses. Tailoring integrated approaches to local environmental conditions is essential to moving toward sustainable, resilient, and climate-responsible viticulture.

## 1. Introduction

Atmospheric CO_2_ concentration has increased over the past few hundred years, since the Industrial and Agricultural Revolution of the 19th century. As the faster-increasing greenhouse gas, CO_2_ plays a central role in global climate change [1]. In agriculture, diverse production systems are being studied to assess their emissions and to identify strategies for mitigation. Within the wine sector, studies span grape production through winemaking, with many emissions directly linked to fossil fuels use for machinery and energy [2]. Agricultural soils are a major terrestrial carbon reservoirs and represent a practical mitigation pathway when managed to increase soil organic carbon (SOC). Recent syntheses demonstrate that regenerative and conservation practices can increase SOC in croplands and vineyards, with implications for climate mitigation, soil fertility, and farm resilience. For the wine sector, SOC gains translate into co-benefits (improved water retention, reduced erosion, enhanced nutrient cycling) but also require evaluation of economic trade-offs and permanence of sequestration under local climates and management [3]. Because SOC stocks are large relative to atmospheric carbon pools, even modest changes in SOC can influence atmospheric CO_2_ a 10% change in SOC has been estimated to be equivalent to several decades of anthropogenic emissions in some assessments [4]. Therefore, small changes or releases of carbon from the soil can lead to considerable changes at the atmospheric level. Intensive agriculture, changes in land use, intensive tillage, and soil erosion have generated SOC losses, contributing 25% to anthropogenic CO_2_ emissions [5].

Soils rich in organic carbon have better structure, fertility, and biological activity [6], resulting in increased resilience to drought. By definition, soil health links biodiversity and ecosystem functioning to the amount of SOC [7].

In addition to ecological aspects, economic factors influence the decision to use practices that improve SOC. This is because improving nutrient cycling enhances agricultural profitability [8]. Comparative studies have found that reduced tillage, the use of organic fertilizers, and cover crops can increase SOC, although the magnitude of the increase depends on various environmental factors, crop type, and the time scale of observation [7].

Low SOC, along with low biological activity, is associated with compaction and higher risk of erosion, accelerating soil degradation. According to a study by the Food and Agriculture Organization of the United Nations (FAO) [9], one-third of soils have suffered degradation, releasing significant amounts of carbon into the atmosphere that could counteract global efforts to mitigate climate change. Eroded soils release even more carbon as crop productivity decreases [10]. When soil is tilled, the top 20 cm are exposed to erosion and carbon loss. It has been estimated that tilled soils can lose up to 50% of SOC within 3–50 years [11], with reported losses ranging from 34% to 82% depending on degradation severity [12]. Soil degradation directly affects nutrient cycles, producing a coupling in the nitrogen and carbon cycles and increasing mineralization, leaching, or emissions processes [5,13,14].

In the wine industry, and particularly in viticulture, mitigation strategies are being developed to both reduce emissions and enhance carbon sequestration. Management practices such as reduced or no tillage [15], organic amendments, and mulches between vine rows are commonly applied to increase SOC and improve its quality [16]. By altering SOC inputs and composition [3,17], these practices provide benefits for long-term sustainability [8]. Sustainable agriculture is promoted worldwide, and soil health is a crucial element. Soil health—defined as the soil’s capacity to sustain essential ecological processes in a resilient and productive manner [18]—is central to these efforts. In other words, soil health represents the interaction between physical, chemical, and biological components, that enables the soil to carry out multiple ecological and productive functions in a continuous and balanced manner. Soil is a living and dynamic system that supports plant growth, regulates water and carbon cycles, filters pollutants, and provides habitat for organisms [6]. Therefore, an integrated strategy combining plant covers (permanent covers, legume/non-legume mix), reduced tillage, and practices tailored to site characteristics (soil texture, climate, slope) offers a promising avenue for simultaneously improving climate change mitigation and soil health in viticulture.

This review explores how vineyard management influences carbon sequestration and soil health. The analysis is structured around (i) carbon storage mechanisms in vineyards, (ii) the role of cover crops, and (iii) their combined effects on soil health and resilience to climate change.

**Figure 1:**
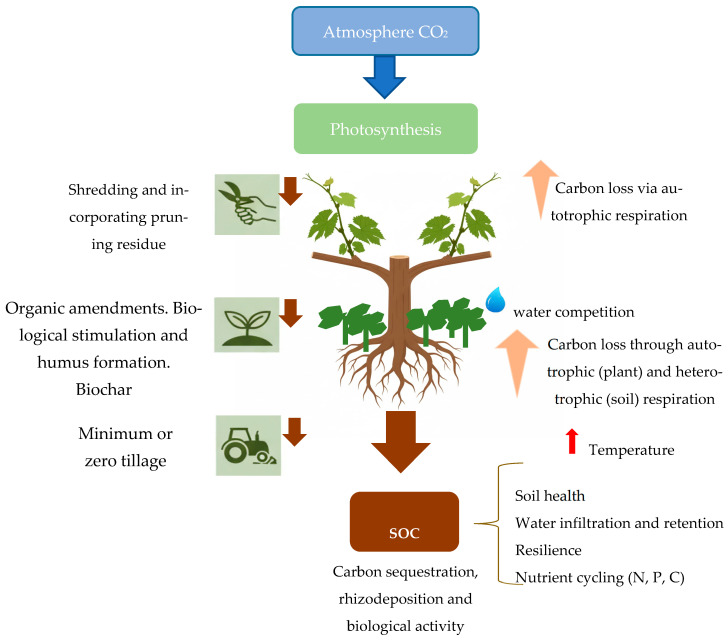
Conceptual model linking vineyard management practices (cover crops, organic amendments, biological stimulation and humus formation, pruning residue management, reduced tillage, biochar) with carbon fluxes in plants and soil (photosynthesis, root resource allocation, rhizodeposition, litter inputs, heterotrophic and autotrophic respiration) and with ecosystem services (soil health, water infiltration and retention, nutrient cycling, resilience). Downward arrows indicate processes that increase soil organic carbon (SOC), while upward arrows indicate processes that release CO_2_. The figure also symbolizes how the presence of plant cover induces competition for water and how temperature influences soil biological activity. This figure is cited throughout the text to illustrate how individual practices influence multiple components of the system.

A conceptual schematic (Figure 1) summarizes the main processes and management levers discussed below.

## 2. Carbon Sequestration in Vineyards

Grapevines are highly adaptable to a wide range of altitudes and climatic conditions [19,20] and are grown under diverse systems such as traditional bush vines or “pergolas”, an elevated training system, with shoots and foliage are trained on a horizontal plane, typically 1.8–2.2 m high. Like all plants, grapevines carry out two antagonistic physiological processes during their vegetative cycle: photosynthesis and respiration. Through photosynthesis, atmospheric CO_2_ is fixed into organic compounds which are used primarily in primary metabolism, forming both the permanent structure and the annual photosynthetic biomass of the plant. The main compounds produced are cellulose and lignin, which accumulate in permanent organs, such as roots, trunk, shoots, buds, and leaves, as well as in the synthesis of sugars in grapes. Beyond primary metabolism, carbon compounds also participate in secondary metabolism, generating compounds such as polyphenols, which accumulate in the berries and contribute to the quality of the wines. The carbon stored in the bunches as structural carbon leaves the vineyard system at harvest. While structural carbon from leaves returns to the soil through litter, non-structural carbohydrates are remobilized and stored in perennial organs such as roots, trunk, and branches to ensure the next growing season [21,22,23]. Roots alone can account for 10–25% of total plant carbon [24]. Reported total biomass carbon stocks vary by cultivar, age, and management; for example, Cabernet Sauvignon vineyards have shown total biomass carbon stocks of up to 12.23 Mg ha^−1^ [20], with roots contributing substantially to this sequestration [24].

Soil organic carbon (SOC) is a vital component of soil health and fertility, while also helping mitigate global warming by reducing atmospheric carbon dioxide (CO_2_) concentrations [25,26]. Organic carbon enters the soil via plant residues, root exudates, and organic amendments, and is retained by management practices that reduce its decomposition or loss. When plant remains (both leaves and roots) is decomposed by microorganisms, carbon enters into the soil organic matter pool. Additionally, plants release organic exudates into the soil, which increase SOC levels. The balance between organic inputs and decomposition determines whether soil organic matter increases or decreases. In vineyards one of the most effective strategies for enhancing SOC is the use of cover crops [23,27,28]. Other practices include the application of organic matter such as compost, manure (7–7.5 Mg CO_2_ ha^−1^ year^−1^), cover crop (2.8–6.5 Mg CO_2_ ha^−1^ year^−1^) and vine pruning (5–8 Mg CO_2_ ha^−1^ year^−1^), and the combination of these practices achieves higher values (11 Mg CO_2_ ha^−1^ year^−1^) [29] which supply substrates that improve soil structure and stimulate microbial activity. Recent studies suggest that the addition of organic fertilizers can significantly increase SOC by up to 40%, with carbon sequestration rates close to 7.5 Mg CO_2_ ha^−1^ year^−1^ [29]. Scientific research is generating information on how sustainable vineyard management can sequester carbon and enhance resilience to stress. The implementation of soil management practices in vineyards has been shown to be effective in enhancing SOC stocks and contributing to atmospheric carbon sequestration (Table 1). The meta-analysis conducted by Payen et al. [29] showed an average SOC sequestration rate of 7.53 Mg CO_2_ ha^−1^ year^−1^ when various carbon sequestration practices were used, although rates vary widely depending on the practices, pruning residues, environmental conditions, and cultivars. Among individual practices, organic matter input has shown a 44% increase in SOC and a sequestration rate of 7.89 Mg CO_2_ ha^−1^ year^−1^, being similar to other multi-annual agricultural systems [7,30]. Biochar is a stable carbonaceous material obtained by heating biomass (such as plant remains, agricultural or forestry waste) in the absence or with very little oxygen, through a process called pyrolysis. Biochar stands out for its ability to improve carbon stability in the soil, reducing decomposition and promoting long-term carbon retention, especially in vineyards. This compound naturally occurs in fire-prone ecosystems such as savannas and grasslands, where it can represent a significant fraction of SOC. Its application in vineyards has been shown to increase SOC by 18%, resulting in an increased sequestration rate of 8.96 Mg CO_2_ ha^−1^ year^−1^ (Figure S1). While this study was short-term, it is critical to assess the sequestration rate and SOC over the long term [31]. The overall carbon balance in vineyards depend on land-use history and timing of assessment. The conversion of uncultivated land into vineyards often entails a significant initial loss of SOC. A comparison between cultivated and uncultivated areas has revealed that the latter have twice or even more SOC.

Another practice that is becoming more general in certain regions of the world is the use of plant cover, which consists of maintaining planted or spontaneously growing vegetation between the rows of vines. Cover crops generally improve soil structure, increase organic matter, enhance water retention, and promote nutrient cycling [44]. When the plant cover is perennial, SOC levels can increase up to 1.4-fold in five years, with a sequestration rate of 4.45 Mg CO_2_ ha^−1^ year^−1^ [30,45,46]. Combining practices often produces synergistic effects higher than the sum of individual benefits. An example of this is the combination of organic fertilization and no-till. Studies have shown that this combination resulted in a 60% increase in SOC and a sequestration rate of 11.06 Mg CO_2_ ha^−1^ year^−1^ [30]. When plant cover (organic fertilization + no-till + plant cover) are added to this combination, SOC increased by 41% and a sequestration of 10.51 Mg CO_2_ ha^−1^ year^−1^ was achieved [47], making it a highly recommended strategy in viticulture. Similarly, the combination of no-till and cover crops resulted in sequestration rates of 7.63 Mg CO_2_ ha^−1^ yr^−1^, 1.7 times more than cover crops under conventional tillage [46]. Traditionally, winter pruning residues were removed from vineyards. Currently, they are cut into small pieces and left on the soil surface or incorporated into the soil profile through tillage. This practice was evaluated individually, and the return of pruning residues recorded the lowest sequestration rate (2.82 Mg CO_2_-eq ha^−1^ yr^−1^) [48], whereas the combination of pruning residues with no-till and plant cover, achieved 6.35 Mg CO_2_-eq ha^−1^ year^−1^ [48].

Carbon sequestration rates also varies with climate. According to the Köppen–Geiger classification, cold semi-arid climates had the highest rate (11.40 Mg CO_2_ ha^−1^ year^−1^), while hot desert climates had the lowest (0.79 Mg CO_2_ ha^−1^ year^−1^), largely due to water and soil limitations [30]. Temperate climates showed intermediate values (7.98 Mg CO_2_ ha^−1^ year^−1^). The duration of study also affects results. Short term experiments (<6 years), report the highest carbon sequestration rates (8.66 Mg CO_2_ ha^−1^ year^−1^); whereas medium-term studies (6–10 years) average 6.95 Mg CO_2_ ha^−1^ year^−1^, and long-term studies (>10 years), show the lowest rates (3.99 Mg CO_2_ ha^−1^ year^−1^). This is likely due to a rapid initial accumulation of SOC followed by an equilibrium point [30]. These data indicate that semi-arid vineyards have lower potential due to high temperatures and limited water availability. High temperatures increase respiration rates and mineralization, while low water availability limits carbon sequestration.

The majority of CS by a vineyard resides in the soil. Zhang et al. [49] found 55.35 Mg ha^−1^ of total carbon in a Cabernet Sauvignon vineyard, with 77.9% in the soil and 22.1% in vine biomass, largely in roots. CS under similar conditions. Song et al. [44] recorded a sequestration of 131 ± 7.1 Mg C ha^−1^ year^−1^ in vineyard during the first years. Similarly, Nistor et al. [35], found total carbon stocks ranging from 8.02 Mg ha^−1^ for white cultivars (Chardonnay) to 42.75 t ha^−1^ for red cultivars (Cabernet Sauvignon), [50]. In different production systems such as Californian vineyards, CS averaged 87.10 Mg C ha^−1^ year^−1^. These results highlight the important role of roots, although knowledge about carbon allocation in perennial grapevine organs is still limited.

The rate of carbon accumulation varies depending on soil depth. The highest carbon concentrations occur in the top 20 cm of soil but deeper soil layers can also store substantial carbon, especially where deep roots or perennial covers are present. In the first 20 cm of Cabernet Sauvignon vineyards, 34% of carbon was sequestered, and up to 40 cm, 62%, decreasing with depth. We therefore recommend depth-resolved sampling (e.g., 0–10, 10–20, 20–40 cm) and standardized reporting to improve comparability among studies. However, studies at greater depths are needed, which could provide more information due to the respiration of deep roots or the greater stabilization of carbon [51,52].

Net SOC sequestration reflects the balance between inputs and outputs (Figure 1). Carbon outputs include autotrophic respiration (root respiration) and heterotrophic respiration (microbial and faunal decomposition), as well as erosion and leaching [53,54,55]. Practices that increase organic inputs (e.g., organic fertilization, cover crops) often stimulate microbial activity and basal respiration, which can raise short-term CO_2_ efflux even as SOC stocks grow; the net outcome depends on stabilization mechanisms such as aggregation and mineral association. Basal soil respiration is an indicator of microbial activity and is directly affected by abiotic factors, inducing non-predictive responses [3]. Microorganisms regulate several processes, including nutrient recycling, carbon stabilization, and the decomposition of organic matter [56].

No-till farming commonly reduces respiration by 4 to 22% compared to conventional tillage [57]. This is because tillage exposes organic matter to oxidation, increases aeration and temperature, thus increasing microbial respiration and releasing carbon into the atmosphere. Excessive nitrogen fertilizer application also increased soil respiration by more than 15% [58]. Other management practices significantly influence the carbon balance too. Brunori et al. [24] compared net carbon fixation in the aboveground and belowground grapevine organs under conventional and organic systems, accounting for root respiration. These authors showed similar carbon sequestration rates between organic (5.38 ± 1.15 Mg C ha^−1^ year^−1^) and conventional vineyards (6.26 ± 1.1 Mg C ha^−1^ year^−1^). However, organic vineyards, which also benefited from soil health through the use of organic fertilization and the minimization of tillage, tended to sequester more soil carbon compared to traditional vineyards by increasing SOC and microbial activity (Figure S1).

Carbon allocation also depends on vine genotype, plant age, and size. Although grapevines accumulate carbon similarly to nearby natural vegetation [59], soil remains the primary carbon reservoir in viticultural ecosystems. For example, allometric studies indicate that when vineyard carbon stocks average 12.3 Mg C ha^−1^, perennial structures are responsible for 8 Mg C ha^−1^ year^−1^, and leaves and fruits each account for approximately 1.7 Mg C ha^−1^, [59]. In Italian vineyards, the root system alone accounts for 9–26% of the total grapevine CS, with a total storage of 5.2–7.2 Mg C ha^−1^ year^−1^ [60].

## 3. Plant Covers in Vineyards

Cover crops play an important role in carbon sequestration. The plant species used as cover crops can be deliberately planted or grow spontaneously between the vine rows. They can also include species adapted to other climates or with species native to the region. When native to the area (planted or spontaneously growing), they also provide multiple ecosystem services. Cover crops enhance carbon sequestration, soil fertility, and biological activity by adding above- and belowground biomass, root exudates, and litter that feed soil organisms and build soil organic matter [29,61]. Spontaneous (native) plants often deliver additional ecosystem services because they are adapted to regional climate and soil conditions, reduce establishment costs and water consumption compared to sown crops and improving soil structure, biodiversity, and terroir expression [24,62]. In general, native vegetation promotes long-term ecological stability and ecosystem services, while sown cover crops optimize short-term production management.

When the vineyards had plant covers, they act as a carbon sink to a greater extent than if the soil is bare, although sequestration rates vary with species and management (Figure S1) [44]. Perennial plants with deep and extensive root systems have the potential to increase CS more than annual plant covers as belowground biomass (roots) can contain between 9% and 26% of total carbon, depending on multiple factors.

Rhizodeposition and fresh organic matter inputs serve as substrates for soil microorganisms, enhancing microbial diversity (including bacteria and fungi, and the relations) and nutrient cycling, particularly for carbon and nitrogen [18,63,64,65]. This biological activity improves pore formation and aggregate stability in the soil [66]. All these benefits increase the resilience of agricultural systems to climate variability and reduce dependence on synthetic fertilizers. Therefore, agriculture plays a dual role in the carbon cycle, acting as a source of greenhouse gas emissions. In general, the most commonly used plant cover species in vineyards include cereals (*Hordeum vulgare*, *Secale cereale*, *Avena sativa*), legumes (*Trifolium* spp., *Vicia* spp., *Medicago* spp., alfalfa), and other species such as phacelia or buckwheat. Selection depends on soil characteristics, water availability, and production objectives [67,68,69]. Table 2 presents different cover crop species and their impact on an agricultural system. Perennial species, with denser and deeper root systems, generally promote more persistent belowground carbon inputs and promote stable microbial communities; all of which improve soil health and long-term carbon sequestration. However, species choice should be tailored to soil texture, water availability, climate, and production goals. For example, legumes supply nitrogen and can improve soil N status, while grasses often produce high biomass and structural residues that favor aggregation [70,71].

These plant covers provide numerous benefits for crops, including grapevines. The main benefits of using plant covers have been determined to be: (1) improved soil fertility and nutrient cycling (through atmospheric nitrogen fixation and increased soil organic carbon), (2) improved soil structure, (3) optimized water infiltration and retention, (4) increased resilience to adverse conditions, (5) weed control, (6) increased plant biodiversity, (7) attraction of beneficial insects, (8) decreased water and wind erosion, among others [68,83].

The benefits of vegetation cover on carbon sequestration also vary depending on the location. Inter-row covers typically contribute most to SOC accumulation across the row middles, while under-vine covers have stronger, localized effects on vine water competition and microclimate.

Table 2 describes the main characteristics and functional advantages of legume cover crops used in vineyard. In the case of fescue cover SOC can reach values of 40–120% compared to bare soil, and only 20% under the plant [30,45,84]. This variation depends on the area covered.

*Trifolium fragiferum* L. is an important cover crop option for soil management in Mediterranean vineyards, as it controls weeds without negatively affecting yield or grape composition. This perennial species can fix nitrogen and cover more than 85% of the surface, eliminating weeds through competition within four years. In irrigated vineyards, *Secale cereale* L. is often preferred because its high biomass improves soil organic matter and reduces nitrogen leaching [71]. This species also tolerates drought and a wide pH range, making it an alternative to herbicides and tillage. In wetter years, mixtures of *T. fragiferum* and *Festuca ovina* have been shown to increase crop yield [85]. White clover and mixed clover species produce higher biomass and nitrogen efficiency, improving nitrogen storage in the soil. *Trifolium incarnatum* grows rapidly, regenerates in winter and spring, fixes nitrogen, and reduces soil erosion [74,86]. *Medicago* spp. are self-seeding annual legumes highly valued in the Mediterranean, in areas with less than 700 mm of rainfall. Depending on the climate and soil, cover crops can reduce, maintain, or even increase yields. In Australia, Medicago combined with *Lolium rigidum* maintained vine production, as *Medicago polymorpha* showed lower evapotranspiration than bare soil [73,75,78]. In semi-arid conditions, rye improves soil moisture and, in irrigated vineyards, is preferred for its drought tolerance. *Festuca arundinacea* is a deep-rooted perennial suitable for diverse soils and sloping or rainy areas, although it can compete with vines [73,75]. *Hordeum vulgare* establishes quickly and is effective against weeds, but is sensitive to low pH and waterlogging, unlike *Avena sativa*, which adapts to a wide range of soils and pH levels [87]. *Lolium rigidum* and *L*. *multiflorum* are also effective for weed and erosion control, but require careful management to avoid competition with grapevines [73,75,78]. *Phacelia tanacetifolia* improves soil aggregate stability and microbial biomass, and in its first year it improved water status and vine vigor, although these effects diminished in the second year [69,82]. Compared to rye, it has a lower C/N ratio and higher biomass, which benefits soil health. In warm regions, *Portulaca oleracea* reduces temperatures in the fruiting zone, improving grape composition (lower sugar content, higher acidity, anthocyanins, and flavonols) and the sensory quality of the wine (floral notes and complexity) [82].

In Mediterranean areas, the following have been recommended for winter: *Avena sativa*, *Hordeum vulgare*, *Vicia saliva*, and *Brassica napus*. Meanwhile, for regions with temperate or humid climates, the following perennials have been recommended: *Festuca arundinacea*, *Lolium perenne*, *Poa pratensis*, *Trifolium repents*, *Lotus corniculatus* and *Medicago lupulina* [32,45,46,70,75,78,80,83,88].

Vegetation cover also modifies the soil microclimate by promoting moisture retention and thermal stability of the soil profile. Soil temperature decreases in summer in soils with cover due to reduced direct solar radiation [89,90]. In addition, vegetation acts as insulation, maintaining temperatures near the soil during cold periods and mitigating the risk of frost by retaining heat during winter [89,90]. At depths of up to 45 cm, temperatures increased by up to 5 °C [90] during winter, while in summer they reached up to 3 °C. However, this situation changes during the budding period when spring frosts are common. In this situation, cover crops reduce soil heating during the day by decreasing the incidence of solar radiation, and at night, they reduce heat loss from the soil to the air near the border, making the soil up to 3 °C warmer than bare soil [90,91]. This suggests that cover crops could serve a dual purpose in relation to frost and heat damage at different times of the year. To manage this trade-off, adopt timed mowing, strip-seeding, or temporary under-vine removal during frost-sensitive periods. The magnitude varies depending on the cover crop species, as species like *Avena sativa* (sweet oat) or *Lolium perenne* (perennial ryegrass), with their high biomass and fine root density, increase infiltration and reduce evaporation, contributing to cooler and more humid climates than bare soils. However, in climates with a risk of spring frosts, cover crops are not recommended during this time of year.

## 4. Permanent vs. Short-Cycle Plant Covers

**Figure 2:**
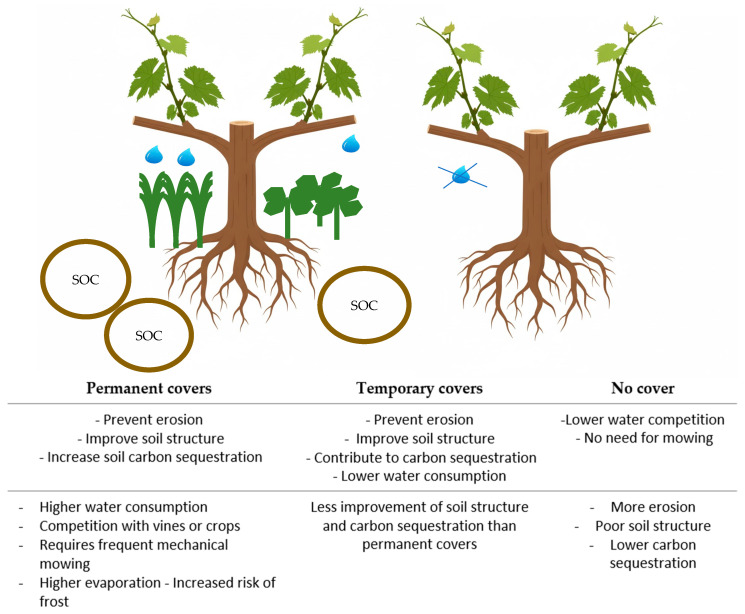
Conceptual model comparing the advantages and disadvantages of permanent, temporary, and no cover. The circles indicate the intensity of carbon sequestration in each situation, and the drops indicate the need for additional water for the grapevine.

Permanent groundcovers provide year-round soil protection, while short-cycle (annual) covers last only a few months (Figure 2) [60,92].

Permanent no-till plant covers significantly increase SOC and total nitrogen [67,73,80,88]. Their continuous presence and the reduction in mechanical tillage improves soil aggregate stability [93,94], allowing faster mechanized access after heavy rainfall for phytosanitary treatments or harvesting [45]. This is particularly important during high-demand periods (e.g., flowering and harvesting).

However, permanent covers may compete with grapevines for water and nutrients, especially in non-irrigated vineyards located in areas with low annual rainfall [78]. To minimize this competition, the growth of the cover should be regulated through strategically timed mowing. These cuts can be complete, in strips or below the plant line [78]. Cover crops can also serve as refuges for beneficial arthropods and natural enemies, thereby enhancing biodiversity, provided species not hosting relevant vine pathogens are chosen [95].

For proper management that does not generate undesirable competition, it is necessary to evaluate the species used, the climate, the soil type, and the phenological state of the vine. Indeed, mowing is a key management practice and should be adapted to the vine’s phenological stage and vineyard conditions [30,45]. It is recommended to mow before the vine blooms. Several objectives are pursued at this time: (1) control competition for water and nutrients; (2) avoid shading the shoots and bunches; (3) allow the cover to re-sprout before summer. In cases where the cover grows vigorously in spring, this first mowing is crucial to avoid affecting bunch set [78]. In summer, when the cover shows excessive growth, it improves the traffic of machinery during veraison or during the application of phytosanitary products. In autumn, mowing favors the establishment of the cover before winter. It is not carried out in all cases, but only in cases where the generated biomass needs to be controlled. In winter, mowing of plant covers controls growth before spring, avoiding risks such as the formation of winter pests. It facilitates entry in spring for the initial phytosanitary treatments.

Interestingly, mowed plant covers can mitigate the risk of late spring frosts by modifying soil surface temperature and radiative balance [96,97,98]. Residues generated by mowing can be left as mulch, conserving moisture and providing organic matter [99].

The effect of plant covers depends strongly on local climate. In areas with annual rainfall exceeding 500–600 mm cover crops induce moderate water stress in vines, which reduces vigor and enhances grape quality.

In general, in these areas, plant cover tends to have the advantage of water consumption, allowing the vineyard to experience moderate water stress, controlling vineyard growth, and improving grape quality (Figure 2) [100,101,102]. Reducing the vegetative vigor of the vineyard can have a positive effect on grape composition, such as reducing titratable acidity and increasing total phenols and anthocyanins in the berry skin. It can also influence the aromatic components of the wine [103,104,105]. However, if at least two harvests are not carried out during the cycle, competition can be excessive and detrimental to the vineyard [36,84]. Plant covers, if not mowed or destroyed before spring, can increase the risk of spring frosts. This is because the mulch limits the accumulation of heat in the soil during the day. Some cover species such as *Vicia faba* or multi-species mixtures can enhance the intensity of frost damage compared to natural grass, as they are denser and tend to trap cold air near the ground [97].

Short-cycle covers, also known as temporary or annual covers, are plant crops that cover the soil for a limited period, usually a single season. Their use has been promoted in vineyards located in regions with greater water availability to improve soil health. Cover crops improve soil health through organic matter inputs, enhance microbial activity, improve structure, and increase resilience.

The species used can be monocultures: *Hordeum vulgare*, *Secale cereale* (for the cold season), *Avena sativa* (with mixtures of other species such as *Vicia faba*, *Pisum sativa*, or *Vicia sativa*), or *Trifolum*, *Lolium rigidum* (for winter-spring growth); *Triticum aestivum*, and often mixed with *Trifolium resupinatum*, *Trifolium michelianum*, *Medicago truncatula*, *Trifolium subterraneum*, and *Medicago polymorpha* [67,83,84].

One of the disadvantages of using plant cover in vineyards is water consumption if the vineyards are located in areas with annual rainfall of less than 500 mm or in semi-arid zones. Therefore, to minimize water consumption by plant covers, the aforementioned strategies are used [106,107,108]. Comparatively, plant covers generally consume more water than bare soil [102]. On average, water consumption by plant covers increases by 0.5 mm more than that of tilled soil between bud break and flowering [109,110]. In terms of evapotranspiration, a vineyard with plant cover has 35% more evapotranspiration than a crop without cover [111]. The annual water required when the crop is covered may need between 2 and 9% as much water. Although in some conditions, no differences have been found [102]. In spring, the surface layer of soil in vineyards with cover dries more quickly and earlier than in vineyards with bare soils because the transpiration of the plant cover is greater than the evaporation of water in the soil [84,112]. This competition can generate increased water stress in the vine, which reduces growth and yields. If the vineyard is irrigated, the use of under-plant plant covers, such as *Trifolium fragiferum*, generates mild water stress without affecting yield or grape composition. In general, it has been observed to reduce water consumption by the vine in post-flowering stages due to lower stomatal conductance and reduced leaf area [113]. In vineyards with deep soils and high rainfall, competition for water in spring reduces excessive vegetative growth of the vine, improving the microclimate of the area of the bunches and consequently the quality of the grapes and the wine [84,114,115].

Under climate change, heat waves and droughts may reduce the carbon sink capacity of non-irrigated vineyards with grassy alleys by lowering Net Ecosystem Production. In short, although ground covers contribute to vineyard sustainability through erosion control and soil carbon sequestration, it is crucial to manage their water use to prevent excessive water stress on vines. Ensuring the economic viability of grape production requires careful species selection; therefore, the use of native plants and appropriate mowing regimes is strongly recommended. Figure 2 shows a comparative summary of the main advantages and disadvantages of using permanent coverage, temporary coverage, and no coverage.

## 5. Soil Health and Resistance to Climate Change

Soil health is directly linked to both the quantity and diversity of microorganisms inhabiting it, which are central to decomposition, stabilization, and nutrient cycling [116]. Key, measurable indicators of microbial function include microbial biomass carbon, basal respiration, and extracellular enzyme activities. Diversified cover crops and organic amendments typically increase microbial biomass and enzyme activity, although they can also raise short-term CO_2_ efflux; long-term SOC gains depend on stabilization pathways such as aggregation and mineral association. The higher microbial in organic systems compared to conventional ones Organic management often supports higher microbial biomass than conventional systems (472 vs. 246 mg C kg^−1^), a difference largely attributable to greater organic matter inputs and reduced chemical disturbance [117,118,119]. Enhanced microbial activity under organic or reduced-disturbance regimes is reflected in elevated enzymes activity involved in the carbon and phosphorus cycles, (β-glucosidase and phosphatase) [120,121,122,123]. These processes increase nutrient availability and soil resilience, contributing to long-term carbon sequestration.

Microbial processes mineralize organic matter and release nutrients that plants can absorb, thereby improving vine nutrition and health [124]. Cover crops, especially legumes, enhances nitrogen availability sometimes reducing the need for fertilization. Mycorrhizal fungi extend root surface area, improving phosphorus uptake, vine photosynthesis and growth [125].

Soil microorganisms are also linked to vine resilience against biotic and abiotic stresses [126,127,128]. Plant can recruit beneficial microbes though root exudates under stress. In sustainable vineyards, collaborative microbial networks favour vine performance under adverse conditions [129,130]. This consequently leads to the establishment of communities of microorganisms that aid the plant in its response to environmental stress [131,132].

Plant-microbe interactions jointly enhance the establishment of the ecological niche for associated microorganisms, improving the growth and immunity of the vine [133]. Furthermore, these beneficial microorganisms prevent the invasion of pathogens. Beneficial genera include *Bacillus*, *Pseudomonas*, *Streptomyces*, *Trichoderma*, *Saccharomyces*, and *Metschnikowia*, which have demonstrated antagonistic properties against common grapevine pathogens, such as the grapevine trunk disease complex (thrush), gray rot (*Botrytis cinerea*), and downy mildew (*Plasmopara viticola*). Beneficial microorganisms perform their function by competing with pathogens for limited soil resources, inhibiting their growth and virulence [134], by influencing nutrient cycling [135,136,137,138]. These microorganisms play a crucial role in improving grapevine health and resilience by activating natural defense mechanisms such as induced and acquired systemic resistance [139,140]. These responses trigger the synthesis of phytoalexins such as stilbenes, phenolic compounds with antimicrobial and antioxidant properties that contribute to the grapevine’s defense system [141,142]. Under drought conditions, microorganisms enhance the grapevine’s ability to cope with stress by regulating osmotic balance, boosting antioxidant activity, and promoting efficient water and nutrient uptake [139,143,144]. In situations of heat stress, the arbuscular mycorrhizal fungus *Funneliformis mosseae* has been shown to increase phosphorus uptake, photosynthetic efficiency, and chlorophyll concentration in grapevines [145]. Phosphorus plays a vital role in plant resistance to heat stress by promoting energy transfer, photosynthetic efficiency, and membrane stability. An adequate supply of phosphorus maintains ATP production, enabling metabolic repair and the regeneration of photosynthetic compounds.

Plant growth-promoting microorganisms (PGMs) further enhance grapevine adaptation by regulating hormonal balance—such as that of auxins, cytokinins, gibberellins, and abscisic acid—and inducing antioxidant enzymes that mitigate oxidative stress [146,147,148,149,150,151,152,153]. These interactions, taken together, strengthen grapevine performance under climatic stress, making soil microbiome management an essential component of sustainable viticulture [122,154]. At the crop level, soils with greater microbial diversity, are associated with more stable and often higher yields over time and in the face of adverse situations such as temperatures and drought [155,156,157]. These results suggest a complex relationship between soil microbial diversity and productive aspects such as crop yield and quality. Factors favouring microorganisms include greater nutrient availability and ecological interactions that promote physiological functions. These effects are amplified under stressful conditions, making it a sustainable strategy for mitigating climate change.

## 6. Conclusions

Innovative vineyard management practices show great potential to mitigate climate change through carbon sequestration and soil health improvement. These practices include the use of plant covers, the incorporation of pruning residues, the use of organic amendments, and no-till cultivation. All of these practices increase the accumulation of SOC stocks, enhance microbial biodiversity, and improve agroecosystem resilience to stress.

The choice of groundcover species and management strategy must be adapted to local soil and climate conditions. In areas with moderate rainfall, permanent covers using native species balance water status, fertility, and nutrient regulation. In contrast, in arid areas, ground cover management must be carefully managed to avoid excessive competition for water and nutrients. Another variable to evaluate is the interaction between the vine and soil microorganisms, such as mycorrhizae and growth-promoting bacteria. These symbioses improve vine stress tolerance, nutrient uptake, and pathogen resistance, providing a sustainable alternative to chemical inputs.

Agroecological vineyard management can thus transform these agricultural systems into important carbon sinks without compromising fruit productivity or quality. Integrating sustainable practices represents a crucial pathway toward climate-resilient, resource efficient, and environmentally responsible viticulture.

Future research should focus on long-term field studies with diverse species, including native, spontaneous, annual, and perennial varieties, integrating soil health and climate indicators (frost risk and heat stress) with economic and environmental outcomes. Furthermore, the development of standardized carbon accounting frameworks and cost–benefit analyses will be crucial to guiding practical implementation in various wine-growing regions.

## Figures and Tables

**Table 1:** Implementation of soil management practices in vineyards (principal strategies).

Vineyard Management Practices	Impact on SOC	Impact on Soil and Water Health	Impact on Vine Productivity/Yield	Other Details/Benefits/ Disadvantages	Authors
Conventional Tillage	SOC losses, 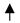 decomposition of organic matter. 52.1% SOC 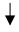 with the conversion of fields to vineyards.	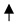 rates of soil erosion, destruction of fungal hyphae and soil fauna habitats	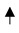 short-term performance	Conventionally emits of 15.4 to 17.41 Mg CO_2_ eq ha^−1^ year^−1^-	Ruiz-Comenero et al. [32]; Coll et al. [33]; Eldon and Gershenson [34]; Nistor et al. [35].
No Tillage	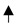 SOC. Sequestration rate of 3.50 Mg CO_2_-eq. ha^−1^ year^−1^. Helps preserve soil aggregates.	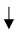 soil erosion, 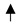 soil structure, and soil moisture. 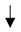 soil respiration. Minimal soil disturbance	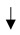 Yield	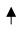 soil carbon. 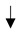 SOC at greater depths.	Eldon and Gershenson [34]; Payen et al. [29]; Visconti et al. [36].
Cover Crops	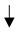 GHG (Greenhouse gas emissions), 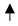 soil fertility/carbon/ SOC (4.45 Mg CO_2_-eq. ha^−1^ year^−1^). 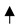 carbon by 1.4 times in 5 years,	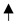 water infiltration rates/soil aggregation/water-holding capacity/arthropod biodiversity/microbial activity 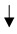 reduce soil erosion. copper phytoremediation	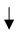 yield (22–85%) for competition.	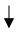 soil surface slow down the decomposition of organic matter, 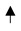 accumulation of SOC	Petersen et al. [37]; Burgio et al. [38]; Nistor et al. [35]; Payen et al. [29]; Visconti et al. [36].
Pruning Residues	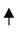 increases SOC.	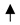 microbial activity/fertility/water infiltration/retention 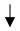 erosion/ nutrient loss optimizing water efficiency.		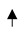 to crush it and incorporate it into the soil carbon sequestration.	Eldona and Gershensonb [34].
Organic Amendments	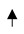 SOC by +44%/ Compost and herbaceous mulch.	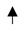 soil fertility and microbial activity/SOC content.			Morlat and Chaussod [39]; Genesio et al. [40]; García-Orenes et al. [41]; Gaiotti et al. [42]; Torres et al. [43].
Biochar	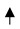 SOC (18%)	=Soil function (Dosage 100 t/ha or more).	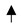 productivity/grape quality.	Most studies are short-term (≤5 years); a long-term evaluation is required.	Paustian et al. [5].

Upward arrows denote an increase, whereas downward arrows denote a decrease.

**Table 2:** Main Characteristics and Functional Advantages of Legume Cover Crops Used in Vineyards.

Cover Type	General Advantages	Species	Particular Advantage	Authors
Legumes	Increases N total and mineral and YAN. Higher N in plant tissues, They produce aerial mass with lower C:N than rye	*Phacelia tanacetifolia*	Increases soil moisture and vigor. Attracts beneficial insects. Improves soil health.	Fernando et al. [69]; Feng et al. [72].
		*Medicago* L.	Loss of yield compared to herbicide control. Self-seeding annuals that are suitable for areas with less than 700 mm of annual rainfall.	Lines et al. [73].
		*Vicia Sativa*	Competes with other species	Nicholas et al. [74].
		*Lotus corniculata*	Can improve the physical structure of the soil by decreasing the bulk density and preventing compaction.	Capri et al. [68].
		*Trifolium*	It adapts to semi-arid conditions with irrigation, controlling weeds effectively. Effective cover crop under the vine in irrigated vineyards, controlling weeds well, with low establishment costs (it is perennial) and providing nitrogen. Multi-cut varieties resprout vigorously after mowing.	Nicholas et al. [74]; Abad et al. [75].
Grasses	It improves soil structure and controls erosion. It prevents leaching. It increases water infiltration, improving soil profile filling in winter. Grapevines are more temperature-resistant than indoor plants, allowing them to grow in the fall after the plant stops growing and before it begins to bud in spring.	*Secale cereale*	Rye can produce a dense cover that is very competitive with weeds. C:N high. Preferred in low rainfall situations. Tolerates dry and poor soils. Tolerates a wide pH range and dry, infertile and sandy soils	Leonard and Andeieux [76]; Nicholas et al. [74]; Kolb et al. [77]; Novara et al. [70].
		*Festuca* spp.	Competing for water. Suitable as a permanent cover between rows (dwarf). Festuca ovina has been noted for having the lowest evapotranspiration (ET) rates among grasses, making it suitable for permanent inter-row cover. Tall fescue can be quite competitive, and complete ground cover with it can reduce yields.	Celette et al. [78]; Capri et al. [68].
		*Lolium*	They are very competitive and fast growing with an extensive fibrous root system.	Nandula, [79].
		*Hordeum vulgare*	It establishes quickly and can produce a dense cover that is very competitive with weeds, useful for erosion control.	Zumkeller et al. [16]
		*Dactylis glomerata*	Can compete excessively with vines. Moderately persistent perennial that tolerates infertile and acidic soils, but not waterlogging.	Nicholas et al. [74]; Abad et al. [75].
Native species (e.g., Wallaby grass, Phacelia, *Atriplex semibaccata*)	They present local adaptation, improve biodiversity, do not impact yield, and in normal years do not require water supplementation. Native species are often the most competitive for both water and nutrients if not managed properly.	*Rytidosperma geniculatum*	Perennial grass native to Australia, well adapted to low humidity and nutrient conditions, without aggressively competing with vines.	Lines et al. [73].
		*Phacelia tanacetifolia*	Improve vine vigor	Ball et al. [80]; Fernando et al. [69]
		*Paspalum vaginatum*	Increased the frequency of earthworms	Lines et al. [73].
		*Atriplex semibaccata*	It works very well as a cover crop in hot, dry environments with high biomass production and weed suppression.	Penfold and Collins [81].
		*Portulaca oleracea*	It reduced photosynthetically active radiation and temperature in the fruiting zone. It resulted in lower total soluble solids (TSS) content in the grapes, higher titratable acidity (TA), lower alcohol content in the wine, and higher TA in the wine. It increased the anthocyanin and flavonol content of the grapes and wines, and improved the wines’ sensory value, especially floral aroma and complexity.	Peng et al. [82].

## Supplementary Materials

The following supporting information can be downloaded at https://www.mdpi.com/article/10.3390/plants14233610/s1. Figure S1. Representation of soil management practices and their environmental impacts in vineyards. The chart compares six practices—conventional tillage, no-tillage, cover crops, pruning residues, organic fertilizers, and biochar—based on their relative effects.
