# Supplementary material for: Carbon Sequestration, Plant Cover, and Soil Health: Strategies to Mitigate Climate Change
Source: Plants (Basel). 2025 Nov 26;14(23):3610. doi: 10.3390/plants14233610 (PMC12694154; doi:10.3390/plants14233610)
Supplement: Supplementary file 1 [file plants-14-03610-s001.zip › plants-3954709-supplementary.pdf]

Supplementary Materials

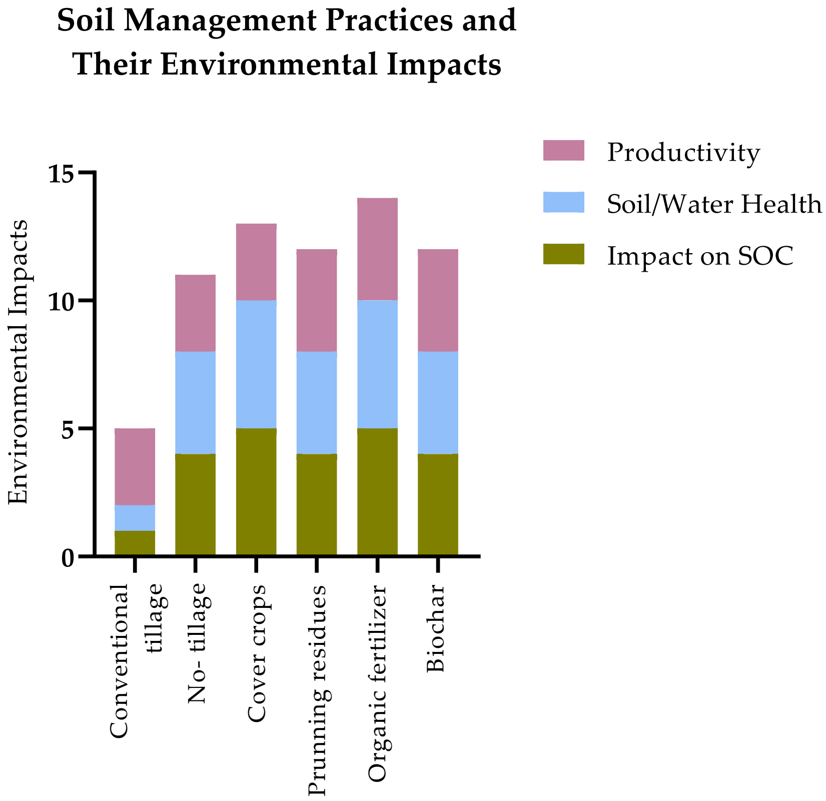

Figure S1. Representation of soil management practices and their environmental impacts in vineyards. The chart compares six practices—conventional tillage, no-tillage, cover crops, pruning residues, organic fertilizers, and biochar—based on their relative effects. Values represent effects reported by various authors for each practice: conventional tillage, no-tillage, cover crops, pruning residues, organic amendments, and biochar. Values reflect the relative effects reported by each study, allowing for a visual comparison of the impacts of different management strategies. Data were compiled from Ruiz-Comenero et al.,[32] Coll et al., [33]; Eldon and Gershenson [34], Nistor et al., [35]; Payen et al., [29]; Visconti et al., [36]; Petersen et al., [37]; Burgio et al., [38]; Morlat and Chaussod [39]; Genesio et al.,[40]; García-Orenes et al., [41]; Gaiotti et al., [42]; Torres et al., [43]; Paustian et al. [5].
